# Two-Photon Functional Imaging of the Auditory Cortex in Behaving Mice: From Neural Networks to Single Spines

**DOI:** 10.3389/fncir.2018.00033

**Published:** 2018-04-24

**Authors:** Ruijie Li, Meng Wang, Jiwei Yao, Shanshan Liang, Xiang Liao, Mengke Yang, Jianxiong Zhang, Junan Yan, Hongbo Jia, Xiaowei Chen, Xingyi Li

**Affiliations:** ^1^Brain Research Center, Third Military Medical University, Chongqing, China; ^2^Department of Urology, Institute of Urinary Surgery, Southwest Hospital, Third Military Medical University, Chongqing, China; ^3^Brain Research Instrument Innovation Center, Suzhou Institute of Biomedical Engineering and Technology, Chinese Academy of Sciences, Suzhou, Jiangsu, China; ^4^CAS Center for Excellence in Brain Science and Intelligence Technology, Shanghai Institutes for Biological Sciences, Chinese Academy of Sciences, Shanghai, China

**Keywords:** two-photon Ca^2+^ imaging, auditory cortex, behaving mouse, dendritic spines, cell-attached recordings

## Abstract

*In vivo* two-photon Ca^2+^ imaging is a powerful tool for recording neuronal activities during perceptual tasks and has been increasingly applied to behaving animals for acute or chronic experiments. However, the auditory cortex is not easily accessible to imaging because of the abundant temporal muscles, arteries around the ears and their lateral locations. Here, we report a protocol for two-photon Ca^2+^ imaging in the auditory cortex of head-fixed behaving mice. By using a custom-made head fixation apparatus and a head-rotated fixation procedure, we achieved two-photon imaging and in combination with targeted cell-attached recordings of auditory cortical neurons in behaving mice. Using synthetic Ca^2+^ indicators, we recorded the Ca^2+^ transients at multiple scales, including neuronal populations, single neurons, dendrites and single spines, in auditory cortex during behavior. Furthermore, using genetically encoded Ca^2+^ indicators (GECIs), we monitored the neuronal dynamics over days throughout the process of associative learning. Therefore, we achieved two-photon functional imaging at multiple scales in auditory cortex of behaving mice, which extends the tool box for investigating the neural basis of audition-related behaviors.

## Introduction

Understanding how the brain works requires the knowledge of neuronal activities at multiple scales in the living brain during behavior. Two-photon imaging (Denk et al., 1990) has been widely applied to investigate brain functions at cellular and subcellular resolution *in vivo*, particularly by using Ca^2+^-sensitive fluorescent indicators (Stosiek et al., 2003; Chen et al., 2013). To maintain mechanical stability for high-resolution imaging, early *in vivo* two-photon Ca^2+^ imaging studies were performed on rodents under general anesthesia. However, anesthesia greatly reduces overall brain activity (Berg-Johnsen and Langmoen, 1992; Cheung et al., 2001), shifts several aspects of temporal processing (Reid and Alonso, 1996), causes synchronization and up-down state oscillations (Steriade et al., 1993; Sanchez-Vives and McCormick, 2000; Volgushev et al., 2006) and alters persistent activity (Major and Tank, 2004). To avoid the unwanted side-effects of anesthesia, *in vivo* two-photon imaging studies in awake and behaving animals have become increasingly important. However, a major difficulty in performing two-photon imaging in awake and behaving mice is the imaging artifacts due to relative motion between the brain and the microscope. There are two general strategies of reducing motion artifacts: mechanical stabilization and *post-hoc* image processing. Mechanical stabilization is of higher importance particularly in the Z-axis, since the imaging artifacts induced by Z-axis motions cannot be corrected by *post-hoc* processing.

In recent years, miniaturized head-mounted optics have been extensively developed for two-photon imaging in awake behaving animals that could be relatively freely-moving (Helmchen et al., 2001; Flusberg et al., 2005; Zong et al., 2017). Alternatively, animals could also be habituated to stay head-fixed and perform behavioral tasks under conventional two-photon microscopes (Dombeck et al., 2007; Gentet et al., 2010; Komiyama et al., 2010). Both approaches have advantages and disadvantages. The miniaturized head-mounted devices allow relatively mechanical-stress-free imaging and thus effectively improve mechanical stability; however, they are more difficult to be fabricated and applied in practical experiments. The major limitation of miniaturized head-mounted imaging devices is the lack of free manipulating space between the brain tissue and the objective, which is required for performing electrophysiological recordings in combination with imaging. On the contrary, using head-fixed habituated animals under conventional two-photon microscopes is highly compatible with electrophysiological recordings. Besides, the application of an objective with a large field of view, a high numerical aperture (NA) and high collection efficiency can be easily achieved. However, the major drawback of head-fixed animals is that they have relatively high stresses and need to be released for comfort after certain duration of time. Considering the above-mentioned issues, two-photon Ca^2+^ imaging studies in awake mice using habituated head-fixed animals have become more favorable and standardized (Greenberg et al., 2014). Combined with an air-supported spherical treadmill to allow walking or running, two-photon imaging in the somatosensory cortex was established in head-fixed awake mice with minimal motion artifacts (Dombeck et al., 2007, 2009, 2010). So far, the target brain regions of two-photon imaging in awake animals have mostly focused on the dorsal surface of the brain, involving the visual (Lee et al., 2016), somatosensory (Dombeck et al., 2007), motor (Dombeck et al., 2009; Komiyama et al., 2010; Chen et al., 2017; Peters et al., 2017), and prefrontal cortices (Low et al., 2014) and the olfactory bulb (Vincis et al., 2012; Chu et al., 2016). However, the lateral brain regions, including the auditory cortex, remain difficult to be accessed by conventional upright two-photon microscopes.

The auditory cortex, which is located in the dorsal lateral region of the brain, is one of the central nervous systems for processing sound information. There have been many studies on the mouse auditory cortex using two-photon imaging in anesthetized mice (Bandyopadhyay et al., 2010; Rothschild et al., 2010; Chen et al., 2011, 2012). For two-photon imaging in anesthetized mice, the auditory cortex can easily fit the upright objective by rotating the mouse body (Chen et al., 2011). However, this method could not be easily applied on awake mice, because it may result in discomfort and struggling. The major difficulty for two-photon imaging in the auditory cortex of awake mice is that there are abundant temporal muscles and arteries around the ears, which makes it difficult to reduce trauma during surgery and which also makes inflammation after surgery likely. Several studies have successfully overcome the difficulty and achieved two-photon imaging in the auditory cortex of awake or behaving mice (Issa et al., 2014; Kato et al., 2015, 2017; Deneux et al., 2016; Kuchibhotla et al., 2017). Some studies (Kato et al., 2015, 2017; Kuchibhotla et al., 2017) used the method of tilting the objective to fit the upright fixed mice. However, the tilted objective might not be easily installed to some conventional microscope systems. Therefore, there remains a need of an alternative approach for performing two-photon imaging in the auditory cortex in head-fixed awake mice, for example, rotating the animal's head (Issa et al., 2014; Deneux et al., 2016). Using acute (Issa et al., 2014) or chronic (Deneux et al., 2016) cranial windows, they have successfully recorded neuronal activities at the cellular level, but it may be difficult to perform electrophysiological recordings and also difficult to achieve sub-cellular imaging.

Two major concerns shall be addressed regarding the surgical preparation for conducting *in vivo* two-photon Ca^2+^ imaging in the auditory cortex. First, the need of combination of electrophysiological recordings with two-photon imaging. Temporal precision is of high importance for auditory processing, but Ca^2+^ imaging alone may not offer adequate temporal precision. Therefore, the head fixation chamber should allow free access and manipulation of micropipettes. Second, proper treatment to the exposed skull for chronic experiments using GECIs (Xu et al., 2012; Goldey et al., 2014; Kato et al., 2015, 2017; Li et al., 2015; Kuchibhotla et al., 2017). For chronic imaging in the auditory cortex, the difficulties in aseptic surgery and recovery were amplified, including skull irregularities, too much covering tissue, and the strict requirements of aseptic conditions. Thus, it remains challenging to perform chronic two-photon imaging in the auditory cortex of behaving mice.

Here, we present an integrated protocol for two-photon imaging in the auditory cortex of behaving mice. This protocol is based on our previous studies (Chen et al., 2011; Li et al., 2017a,b), and the surgical procedures are summarized from hundreds of performances. We optimized the method of the rotated head fixation by using a custom-made head fixation apparatus. First, the custom-made head-post was cemented at the dorsal region of the skull, instead of around the cranial window as in previous studies (Issa et al., 2014; Deneux et al., 2016). Second, the recording chamber was glued around the cranial window separately. It was filled with liquid medium, artificial cerebral-spinal fluid (ACSF), for electrophysiological recordings. Third, a water-restricted procedure was used to accelerate the habitation of head fixation. Fourth, standard aseptic surgery was performed to reduce the risk of infection. Based on these preparations, we successfully achieved stable two-photon imaging in the auditory cortex of behaving mice. By using synthetic Ca^2+^ indicators, we were able to monitor Ca^2+^ transients at the levels of neuronal populations, single neurons, dendrites and single spines in the auditory cortex during behavior. Moreover, we demonstrate that cell-attached recordings and two-photon Ca^2+^ imaging could be performed simultaneously. Furthermore, the combination of this technique with the GECIs (Chen et al., 2013) allowed for monitoring the activities of individual neurons during the whole process of associative learning.

## Materials and methods

### Animals

C57BL/6J male mice (8–12 weeks old) were obtained from the Laboratory Animal Center at the Third Military Medical University. All experimental procedures were conducted in accordance with animal ethical guidelines of Third Military Medical University Animal Care and Use Committee. Before head-post implantation, mice were socially housed under a 12 h light/dark cycle (lights on at 7 a.m.) and provided with *ad libitum* feeding.

### Auditory stimulation

As described in our previous studies (Chen et al., 2011; Li et al., 2017a,b), the sounds were broadcasted by a free-field ES1 speaker with an ED1 electrostatic speaker driver (Tucker Davis Technologies, USA). The broadband noise (BBN, bandwidth 0-50 kHz, duration 50 ms) was generated by a custom-written software based on LabVIEW 2014 (National Instruments, USA) and transduced to analog voltage by a PCI 6731 card (National Instruments, USA). During experiments, the speaker was put at a distance of ~6 cm to the left ear of the animal. We used a microphone at a distance of ~6 cm to measure the sound levels of the speaker. We calibrated all sound levels with a ¼-inch pressure prepolarized condenser microphone system (377A01 microphone, 426B03 pre-amplifier, 480E09 signal conditioner, PCB Piezotronics Inc, USA). We sampled the data at 1 MHz via a high-speed data acquisition board USB-6361 from National Instruments and analyzed them using a custom-written software in LabVIEW 2014. The BBN was applied at ~65 dB sound pressure level (SPL). The background noise (~55 dB SPL) was below the BBN stimulation level. Further information about the frequency components of the background noise could be found in our previous study (Li et al., 2017a).

### Training procedure

**Figure 1:**
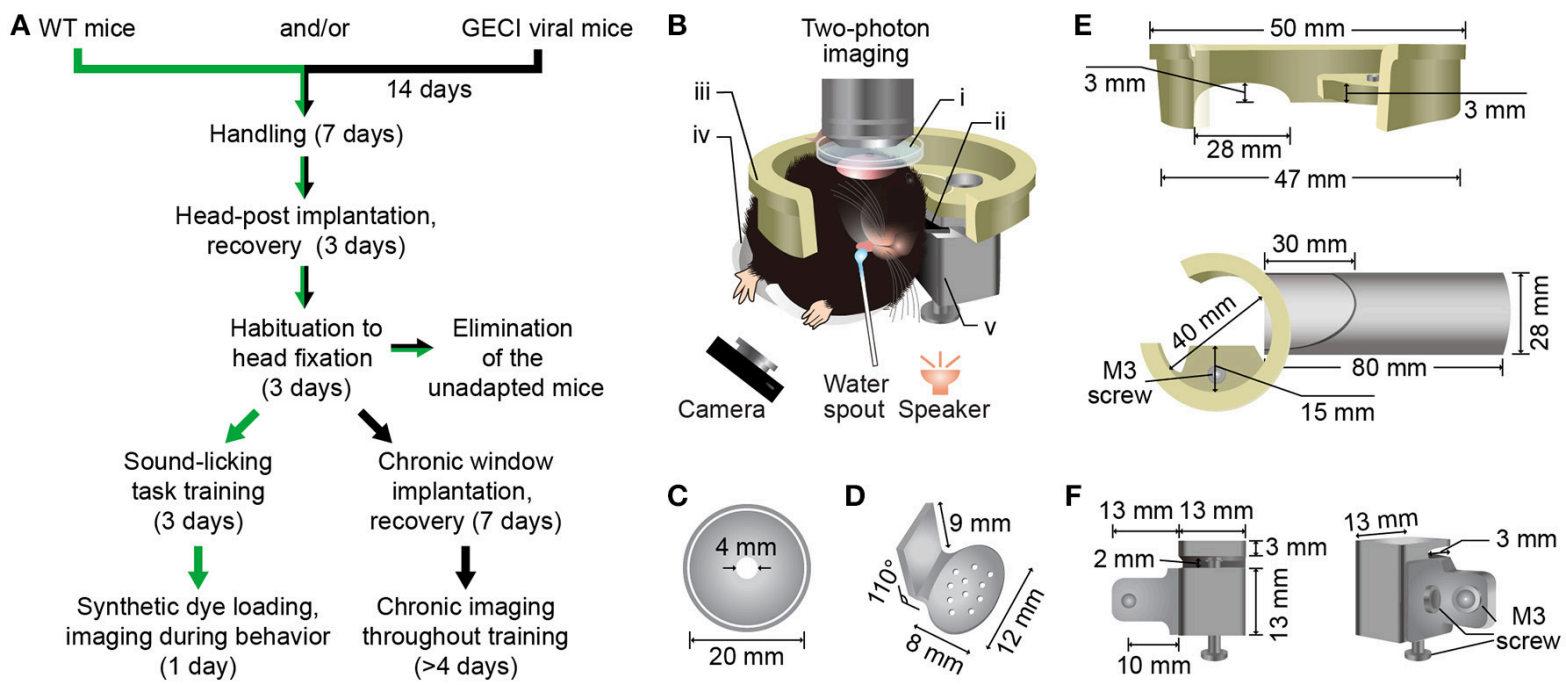
Protocol and custom-made head fixation apparatus for two-photon Ca^2+^ imaging in the Au1 of behaving mice. **(A)** Technical flowchart for two-photon imaging during a sound-triggered licking task. Green or black arrows indicates the route for imaging with synthetic Ca^2+^ indicators or genetically encoded Ca^2+^ indicators, respectively. **(B)** A schematic diagram of a mouse in custom-made head fixation apparatus, performing two-photon imaging during sound-triggered licking task (side view). The speaker was placed at 6 cm below the left ear of mouse and the camera was monitoring the licking behavior at infrared mode. i, Recording chamber **(C)**; ii, Head-post **(D)**; iii, Recording holder (**E**, upper); iv, Body tube (**E**, lower); v, Head-post holder: **(F)**. The detailed design and size of these parts are present below. **(C)** Plastic recording chamber (upper view). The chamber was fit for both electrophysiology and two-photon recording. **(D)** Titanium head-post for head fixation. Consist of two parts with an angle of 110° The lower part has a suitable radian for fitting the skull and several built-in holes for spreading of glue. Another part fits the head-post holder. **(E)** Resinous recording holder connecting the head-fixation apparatus and the microscope. The upper panel shows the fine design fitting the custom-made slot on the objective table of the microscope. The lower shows the connection of the holder and the plastic body tube. The upper part of tube was cut off for unhindered body. The lower part was kept for front paws. **(F)** Head-post holder (stainless steel) was the connective piece for connecting the recording holder, head-post and the body tube with three M3 screws. It consists of two parts. The left part connecting the body tube and the right part connecting recording holder and head-post (left).

The diagram and precise sizes of the head fixation apparatus were presented in Figure 1. It consists of five parts (see Supplementary Files 1–5 for 3D print files). The recording chamber (Figure 1C) is used for containing the liquid medium, ACSF, and protecting the cranial window. The head-post (Figure 1D) is used for the fixation of the mouse head that is rotated ~70 degrees to the left. The recording holder (Figure 1E, upper) is used to link the fixation apparatus with the imaging setup. The body tube (Figure 1E, lower), which was modified from the previous study (Guo et al., 2014), is used to sustain and restrict the mouse body. And the head-post holder (Figure 1F) is used to connect the recording holder and the body tube, and to hold the head-post.

Before head-post implantation, mice were handled more than 10 min per day for 1 week (Figure 1A). After head-post implantation (Supplementary Figure 1), mice were sent back to their home-cage for 3 days' recovery. In addition, we put the body tube (Figure 1E, lower) in the cage and allowed the mice to play with. After recovery, mice were habituated to head fixation, which should last for 3 days. Mice were head-rotated (~70°) and fixed in the custom-made head fixation apparatus (Figure 1B), with free drinking for 1 h every 12 h in the training environment including the body tube, background sounds, training apparatus and experimenters. Then, the mice were released back to the home-cage for water restriction.

**Figure 2:**
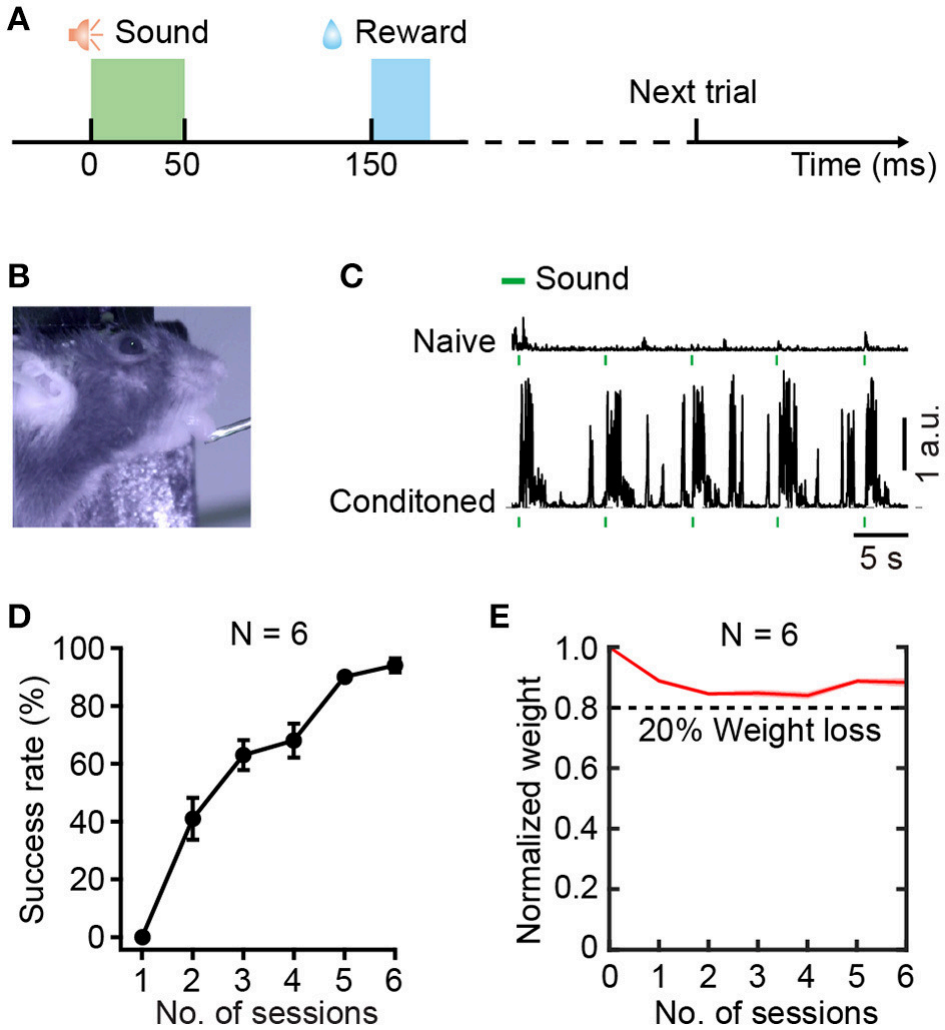
Training for the sound-triggered licking task based on water restriction. **(A)** Schematic of the event timing for each trial of training. **(B)** A head-fixed mouse performing the licking task. **(C)** Licking behaviors before (upper) and after learning (lower), quantified by tongue movements. **(D)** Average success rate of task performance across 6 training sessions over 3 days (*N* = 6 mice). **(E)** Normalize weight change across 6 training sessions over 3 days (*N* = 6 mice). The dashed line indicates 20% weight loss.

Rodents generally perform better with water restriction than food restriction (Treichler and Hall, 1962). Thus, we designed a sound-triggered licking task with water restriction. To minimize interference of visual information, a dark background was needed during training. The licking behaviors were monitored by an infrared camera (30 Hz frame rate). Each trial included a stimulus period (50 ms), an interval between stimulus and reward (100 ms), a water delivering period (20 ms), and an inter-trial interval (ITI) (Figure 2A). Mice were conditioned to associate the BBN stimulus with the water reward. For the success trials, ITI was set randomly between 4 and 6 s. For failure trials, there were no punishment with false alarms, but the ITI was set randomly between 10–12 s.

The relative positions of the lick-port and the mouse is a critical parameter during training (Guo et al., 2014). To avoid compulsive licking, we adjusted the distance between the lick-port and the lower lip from the initial 0.5 mm to the final 4 mm during training. Typically, the lick-port was centered on the midline of the mouse lip and 3 mm posterior to the tip of the nose. At this distance, the tongue of a mouse could just touch the lick-port. Moreover, the behavioral test of each session needed 4 mm below the lower lip to minimize the tactile sense interference of the whiskers. The training included two sessions every day, with an interval of 6–8 h. One session included 150 trials that were divided into 3 groups. There was a break for at least 5 min following each group of trials. According to previous studies, the minimal daily water intake for a 25-gram mouse is ~0.625 ml (Andermann et al., 2010), and the normal daily water demand is ~5.8 ml (Bachmanov et al., 2002). In the first day of training, as most trials were not targeted, the water gained from licking was less than the minimum amount, and a supplementary amount of 0.5 ml water was provided after training. After two sessions of training, the mouse could remember the location of the lick-port. Considering that over half of the trials were targeted in the following 2 days mice that performed 300 trials daily would receive 0.75–1.5 ml of water, no supplementary water was provided. Generally, 6 sessions were sufficient for reaching a conditioned performance, with a success rate over 80%.

### Expression of GECIs

Adult mice were anesthetized with 0.8–1.5% isoflurane in pure oxygen (0.5 L/min) and placed in a stereotactic frame with a heating pad (37.5–38°C). Lidocaine (2%, ~100 μl) was administered locally, and eyes were protected with ointment (Bepanthen, Bayer, Germany). Mice were kept at a relatively constant depth of anesthesia, characterized by the loss of reflexes (e.g. tail pinch) and respiration rates of 80–110 breaths per minute (BPM). The scalp was incised with ophthalmic scissors. For AAV2/8-GCaMP6f (~1 × 10^13^ vg/ml, Shanghai Tai Ting Technology Corp., Ltd, China) injection, a craniotomy (1 mm diameter) was made with a dental drill above the dorsal auditory cortex (AuD, AP: −3.1 mm, ML: 3.8 mm; angle: 65° to horizontal plane, **Figure 7A**). The virus was delivered through a small glass electrode with a tip diameter of 20–30 μm. The tilted electrode was slowly inserted below the AuD pia surface and moved 1.3 mm forward to target the Au1 cortical layer 2/3 (cortical depth in Au1 ~0.3 mm) to infuse virus (20 nl per min). A total volume of 80 nl of virus was slowly injected into the target area in 4 min. To avoid backflow of the virus, the electrode was held in place for 10 min before slowly retracting it. After injection, the ~1 mm cranial window was filled with bone wax and the scalp incision was closed using tissue glue (Vetbond, 3M Animal Care Products, USA). Mice were administered meloxicam (1 mg/kg, Boehringer Ingelheim, Germany) once a day for 2 days to reduce inflammation. After virus injection for 14 days, the mice underwent head-post implantation and behavioral training for chronic two-photon imaging experiments.

### Implantation of the head-post

A custom-made titanium head-post (0.9 g, Figure 1D) was implanted above the dorsal surface of the skull by aseptic operations. The mouse was anesthetized and prepared as described above, followed by removing hair using sterile cotton-tipped applicators with hair removal cream, resecting of skin and clearing of skull fascia (Supplementary Figure 1). Using cyano-acrylic glue (UHU, Germany), the head-post was glued to the skull such that its right lateral edge was < 3.5 mm from the midline and the anterior edge was < 3.0 mm from the bregma. The mouse was separately housed for 3 days to recover with freely available water and was provided with meloxicam (1 mg/kg) once a day. After habituated to the fixing apparatus, the head-posted mouse was sent to the behavior training stage.

### Acute craniotomy and multi-cell bolus loading

Before craniotomy, the mouse was anesthetized and prepared as described above, and the body temperature was kept at 36.5–37.5°C throughout the experiments. After the skin and muscles above Au1 were removed, the recording chamber (Figure 1C) was glued to the skull with cyano-acrylic glue, and a small craniotomy (~2 mm in diameter; center stereotactic AP: −3.0 mm, ML: 4.5 mm) was performed after glue concretionary. The craniotomy was filled with 1.5% low-melting-point agarose, and then transferred to the two-photon imaging setup with a reduced level of anesthesia (0.6% isoflurane, breath rate 90–120 BPM). The recording chamber was perfused throughout the experiment with normal ACSF as in the previous studies (Chen et al., 2011; Li et al., 2017a). The fluorescent Ca^2+^ indicator Cal-520 AM (AAT Bioquest, USA) (Tada et al., 2014) was used for multi-cell bolus loading in the Au1. The loading procedure was performed according to previous studies (Stosiek et al., 2003; Garaschuk et al., 2006), and Cal-520 AM was prepared to obtain a final concentration of ~567 μM. Ca^2+^ imaging was performed 2 h after dye diffusion when the animal was awake, and it could last for up to 8 h.

### Single-cell electroporation

To label the dendrites and spines of Au1 neurons, we used single-cell electroporation as detailed in a previous study (Judkewitz et al., 2009). Borosilicate electrodes with a high resistance (12–15 MΩ) were filled with Ca^2+^ indicators and mounted onto an electrode holder attached to a micromanipulator (Luigs & Neumann, Germany). Before being applied into the electrode, Oregon Green 488 Bapta-1 potassium salt (Molecular Probes) was dissolved in intracellular solution to obtain a final concentration of 1 mM. The neurons of interest were targeted using the “shadow patching” technique (Kitamura et al., 2008). Voltage pulses for electroporation were generated with a series of stimuli triggered by a Master-8 (A.M.P.I, Israel) and were delivered to the neuron by the MVCS-01 iontophoresis system (NPI Electronic, USA). The Ca^2+^ indicator was loaded into neurons by 100 rectangular pulses (−10 V, 0.5 ms duration at 50 Hz). Multiple neurons could be electroporated with the same electrode.

### Cell-attached recordings in behaving mice

As described in previous studies (Kitamura et al., 2008; Chen et al., 2011, 2012), the shadow-patching procedure was applied for cell-attached recordings of single neurons in the Au1. In brief, the neurons of interest were identified by using population imaging simultaneously with behavior. Then, an electrode (5–8 MΩ) was filled with 100 μM Cal-520 potassium salt (AAT Bioquest, USA) dissolved in the extracellular solution containing 125 mM NaCl, 25 mM NaHCO_3_, 2.5 mM KCl, 1.25 mM NaH_2_PO_4_, 1 mM MgCl_2_, 25 mM glucose, and 2 mM CaCl_2_. Recordings were performed with an EPC-10 amplifier (HEKA Elektronik, Germany). Electrophysiological data were filtered at 10 kHz and sampled at 20 kHz using Patchmaster software (HEKA Elektronik, Germany).

### Implantation of chronic cranial window

Standard aseptic procedures were used for surgery. The mouse was anesthetized and prepared as described above. A recording chamber (Figure 1C) was glued to the skull. A craniotomy (1.5 mm in diameter) was made over the right Au1 after the glue concretionary. The surrounding bone of the cranial window was polished to an optimal size for imbedding a coverslip. A double-layered coverslip, which consisted of a minor coverslip (1.5 mm in diameter) attached to a larger one (2.5 mm in diameter) using ultraviolet cured optical adhesives (Norland Products Inc., USA), was embedded and sealed with dental acrylic. The smaller layer fit snugly into the craniotomy, and the larger one was attached to the polished bone. Prophylactic injections of antibiotics (Cefazolin, 500 mg/kg, North China Pharmaceutical Group Corporation, China) were administered before surgery until 3 days after surgery. The mouse was separately housed for 7 days' recovery with freely available water and were provided with meloxicam (1 mg/kg) once a day for 3 days.

### Two-photon Ca^2+^ imaging

The two-photon microscope was similar to that described in previous studies (Jia et al., 2010, 2014; Li et al., 2017a,b). The excitation light with a wavelength of 920 nm was generated by a mode-locked Ti:Sapphire laser (model “Mai-Tai DeepSee,” Spectra Physics, USA). The laser power delivered to the objective could be adjusted from 0 to 120 mW according to the depth of the focal plane. A 40 × /0.8 NA water-immersion objective (3.5 mm WD, Nikon) was used for imaging. For somatic Ca^2+^ imaging, images were acquired at 600 × 600 pixels at a 40 Hz frame rate, and a typical field of view was 300 × 300 μm in size. For dendritic and spine Ca^2+^ imaging (Chen et al., 2011), we used the LOTOS procedure. The scanner of LOTOS-based imaging was changed to the following mode: 500 × 500 pixel image frames at a rate of 40–200 Hz with a varying size of the field of view (maximum 50 × 50 μm). There are 3 tips that might be helpful for minimizing the brain motions. First, the recording chamber (Figure 1C) and the lower part of the head-post (Figure 1D) could be manually modified to fit the skull for tight adhering. Second, to reduce the brain motion, a smaller craniotomy would be better. Third, a coverslip (5 mm in diameter) for the cranial window was placed over agarose and sealed by dental cement after dye loading. The licking behaviors were monitored by an infrared camera (30 Hz frame rate).

### Data analysis

All the data were analyzed offline by custom-written software in LabVIEW 2014, Igor Pro 5.0 (Wavemetrics Inc., USA) and MATLAB 2016b (MathWorks, USA).

To evaluate the performance of the mice in the sound-triggered licking task during training, the tongue movements were measured based on the video with custom-written LabVIEW software and the success rate of the licking response was quantified accordingly. The detection window for successfully licking during data analysis is set as 500 ms. In detail, a region of interest (ROI) was manually drawn in the video frame over the mouth and the water spout. The licking strength was calculated as the frame-by-frame difference of the image intensity of the ROI covered the mouth region of the mouse, hence the “arbitrary units” is just a relative unit of measurement to represent the amount of intensity change.

As previous studies described (Li et al., 2017a), the individual neurons in two-photon imaging data were identified visually, the ROIs for each neuron were drawn manually and the Ca^2+^ signal of each ROI was calculated as the relative fluorescence change Δf/f = (f-f_0_)/f_0_ over time. The f_0_ was estimated as the 25th percentile of the fluorescence values for each ROI. Detection of Ca^2+^ transient was performed based on thresholding criteria about peak amplitude and rising rate. We defined a noise level as 3 times the standard deviation (SD) of the baseline activity.

For correcting brain motions resulting from movements during mouse behavior, we used the image alignment software TurboReg (ImageJ, NIH, USA). The brain motions of mice can be decomposed into motions that parallel to the focal plane (XY-plane) and perpendicular to the focal plane (Z-axis). However, Z-axis motion cannot be corrected offline, and the improvement of offline correction was resulted in the XY axis motions. Frame-by-frame alignment of the imaging data was performed with a translation algorithm (Supplementary Video 1 and Supplementary Figure 2). The brain motions were quantified by mean frame to frame in plane (XY) Euclidean distances. For naive mice (4 mice, 2 focal planes for each mouse, 60 s imaging time for each focus), the motion was 2.1 ± 0.9 μm (mean ± SD). After the same group of mice was habituated to head fixation, the motion was 1.9 ± 1.2 μm, and the brain motions larger than 4.5 μm were significantly reduced (Supplementary Figure 3). Furthermore, the brain motion of the conditioned mice was 1.1 ± 0.6 μm (4 mice, 66 behavioral trials in total).

For the reconstruction of the neuron morphology, Z-stack fluorescent images of a single neuron were projected into an averaged image by using projection software (ImageJ). Based on the averaged projections, a schematic morphology of the single neuron, including soma and dendrites, was manually drawn in Adobe Illustrator CS6 (Adobe Systems, USA).

## Results

### Overview

The procedures for two-photon imaging during the sound-triggered licking task are provided in Figure 1A. Animal preparations were started with handling, followed by implantation of the head-post and habituation to the training apparatus. There were 4.4% mice (6 out of 137 mice failed) that could not adapt to the head-rotated head fixation procedure. Once a mouse was habituated, it was sent to either the acute or chronic experiment stage. For acute experiments, we performed population imaging and sub-cellular imaging separately. The head-fixed mice could be appropriate for two-photon Ca^2+^ imaging of population neurons in the auditory cortex during the sound-triggered licking task. The stable imaging could last for 4–6 h. Using single-cell electroporation, we investigated neural activities at subcellular level simultaneously with behaviors. For the subcellular imaging or cell-attached recordings, the stable imaging of one focal plane could last for 0.5–1 h. For chronic experiments, a chronic cranial window was implanted over the Au1 of the mouse, followed by 1 week's recovery, and then the neuronal activities were monitored during licking throughout the process of associative learning.

### Behavioral training

Head-fixed mice were trained to associate the special sound cue, BBN, with a water reward in the sound-triggered licking task (Figure 2). According to the protocol of behavioral training (Figure 2A), 100 ms after the end of a BBN cue, a droplet of water (~5 μl, duration 20 ms) was delivered to a water spout as a reward in each trial. Figure 2B shows a mouse during a trial of the sound-triggered licking task on the behavioral training setup. Using custom-written software in LabVIEW 2014, we tracked tongue movement during licking. Tiny tongue movements were observed during licking in the first session (upper, Figure 2C), and significant movements were recorded in the final session (lower, Figure 2C). The performance of the sound-triggered licking task showed significant improvement in 6 mice across the 6 training sessions over 3 days (Figure 2D). In the first session, the mice merely responded to the sound cue and seldom performed licking. However, most mice reached a stably conditioned performance (>80% success rate) after 6 training sessions, which meant that mice successfully associated the sound with the water reward. Body weight is an important factor in behavioral training of mice. The individual body weight dynamics (Supplementary Table 1) and the normalized values (Figure 2E) of the 6 mice during the training session showed a slight reduction and then increased. The mice with more than 20% weight loss (less than 5% of the total animals used) were excluded and sent back to their home-cage for recovery with free drinking and would be used in other experiments afterwards. However, a 10% weight loss was usually needed for mice to be motivated to perform the licking task for a large number of trials (Guo et al., 2014).

### Population imaging in behaving mice

**Figure 3:**
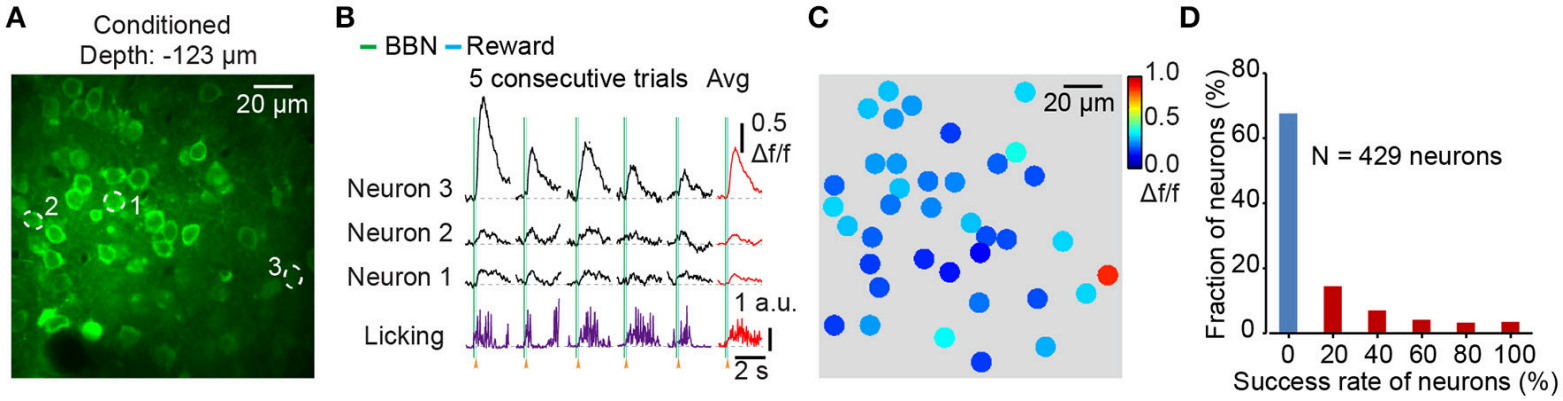
Figure 3. Population imaging in the Au1 of conditioned mice during behavior**. (A)** Average image (400 frames) of a focal plane in layer 2/3 of the Au1. **(B)** Ca^2+^ transients and corresponding licking responses from 5 consecutive trials. Neurons are outlined by dotted circles in **A**. **(C)** Color mapping of the average Δf/f of 3 consecutive Ca^2+^ transients evoked by BBN for each neuron in **A**. **(D)** The fraction of success rate for all neurons in 5 consecutive trials. 429 neurons from 7 conditioned mice.

By using the synthetic Ca^2+^ indicator Cal-520 AM, we could stably record neural responses at the cellular level with a high signal-to-noise ratio. The mouse was recovered enough for behavioral testing after dye loading for 2 h. Figure 3A shows an imaging field in Au1 from a conditioned mouse during behavior and the neurons are outlined by dotted circles. The Ca^2+^ transients of the neurons and simultaneous licking responses are also shown (Figure 3B). Color map (Figure 3C) showed the average Δf/f values of 3 consecutive trials for each neuron in Figure 3A. Among the 429 neurons from 7 mice, 68% neurons were not responsive to the BBN stimulation at all. And the distribution of the success rate was showed in Figure 3D. The success rate was calculated from the response number in 5 consecutive stimuli. Furthermore, since we achieved stable imaging with high frame rates, it provided a possibility to investigate the response latency after training. For example, some Ca^2+^ transients of neuron 1 in a conditioned mouse (Figure 3B) showed a delayed onset which was more closely tied to the reward than the BBN.

### Cell-attached recording in behaving mice

**Figure 4:**
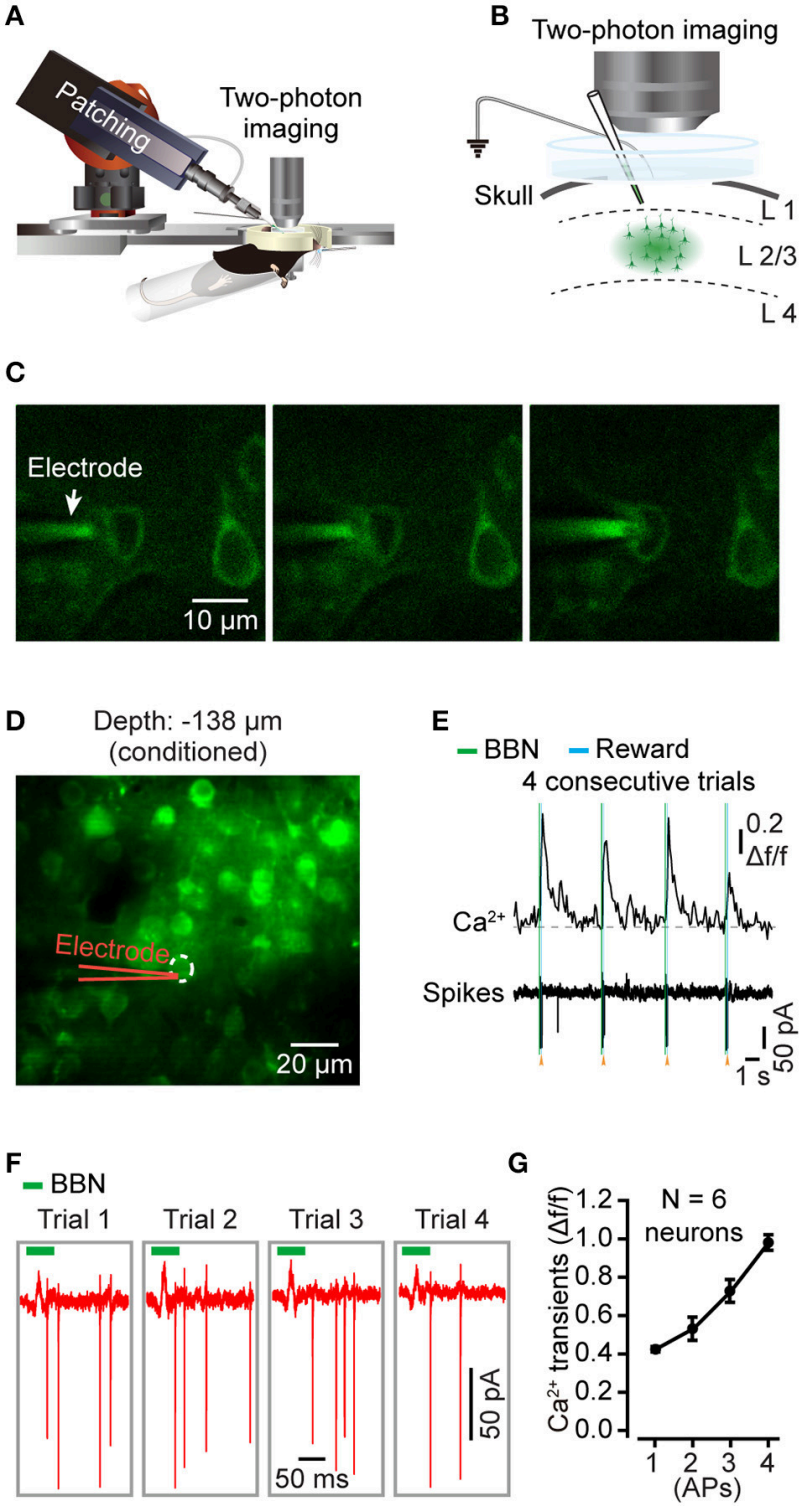
Targeted cell-attached recording of single neurons in the Au1 of behaving mice**. (A)** Schematic of cell-attached recording in the Au1 of behaving mice. **(B)** Zoomed in of the targeted cell-attached recording among neurons labeled by bolus loading in layer 2/3 of the Au1. The electrode was filled with Cal-520 potassium salt (100 μM). **(C)** The process of targeted cell attachment. Left, approaching the targeted neuron. Middle, applying positive pressure to the neuron. Right, sealing the neuron by sucking. **(D)** Average image (450 frames) of a focal plane in layer 2/3 of the Au1. **(E)** Four consecutive trials of sound-evoked Ca^2+^ transients and the corresponding electrical signals from the neuron indicated in **D**. **(F)** Zoomed in of the action potentials (AP) in **E**. **(G)** Comparison of the amplitudes of Ca^2+^ transients associated with different numbers of APs (46 trials of 6 neurons from 4 mice).

We then characterized the action potential (AP) associated Ca^2+^ dynamics among the layer 2/3 pyramidal neurons in the Au1 of behaving mice. Combining two-photon Ca^2+^ imaging and cell-attached recordings (Figures 4A–C), we obtained electrophysiological signals in a conditioned mouse. Using bolus loading, we identified the neurons of interest for the next electrophysiological recordings (Figure 4D). After successful cell-attachment, a neuron showed a string of APs in 4 consecutive trials (Figure 4E). The Ca^2+^ transients and corresponding electrical signals from the neuron (indicated in Figure 4D) showed a high correlation to the sound stimuli (Figure 4E). The zoomed in of the firing action potentials indicated that the number of APs was correlated with the amplitudes of the Ca^2+^ transients (Figure 4F). Statistics showed that the amplitudes of the Ca^2+^ signals and numbers of associated APs were almost linearly correlated (Figure 4G, 46 trials of 6 neurons from 4 mice).

### Subcellular resolution imaging in behaving mice

**Figure 5:**
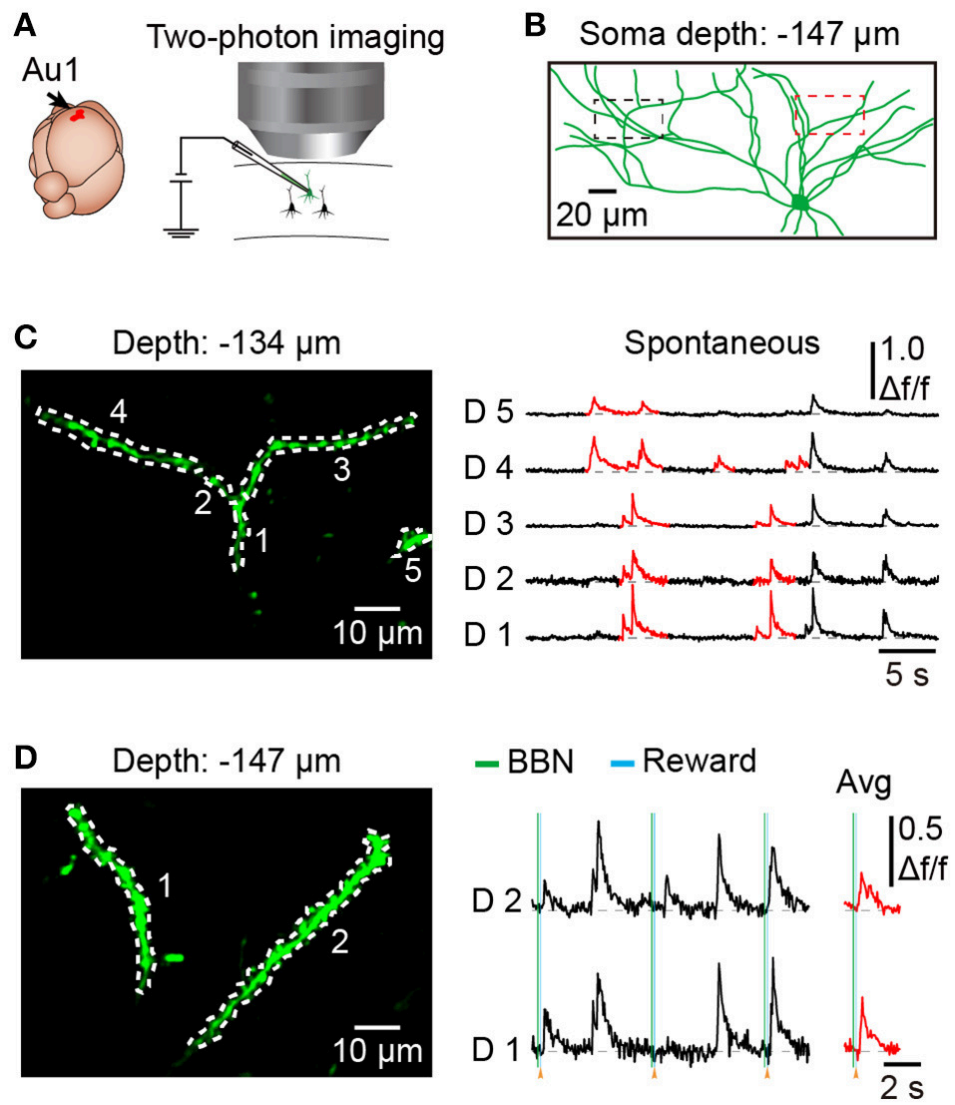
Dendritic Ca^2+^ imaging using electroporation in the Au1 of behaving mice. **(A)** Schematic of electroporation in the Au1. **(B)** Reconstruction of an electroporated neuron in the Au1, labeled with OGB-1. **(C)** Spontaneous Ca^2+^ transients from 5 dendritic shafts (outlined in left part of **B**). Local Ca^2+^ transients are marked in red. **(D)** Dendritic Ca^2+^imaging from a region of the neuron during behavior (outlined in right part of **B**). Average Ca^2+^ transients related to the BBN are marked in red.

**Figure 6:**
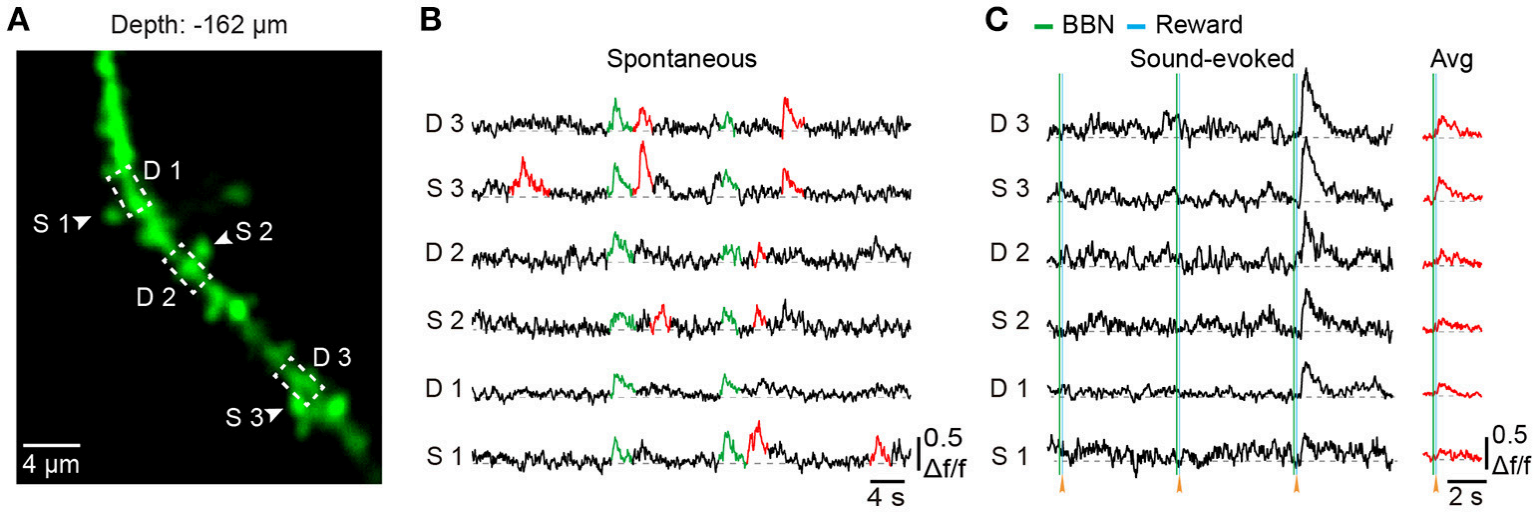
Spine Ca^2+^ imaging using electroporation in the Au1 of behaving mice**. (A)** Average image (1,800 frames) of a dendritic segment. **(B)** Spontaneous Ca^2+^ transients of 3 spines (marked with arrowheads in **A** and their corresponding dendritic shafts (outlined with a dashed rectangle in **A**. Green indicates the assembly of dendritic inputs, and red indicates the single-spine inputs. **(C)** Three consecutive trials of sound-evoked Ca^2+^ transients from the same spines and dendritic shafts as in **B**.

To explore dendritic and spine Ca^2+^ transients of a single neuron in the mouse Au1, we used single-cell electroporation to label a targeted layer 2/3 neuron (Figure 5A). By using conditioned mice and offline motion correction, we obtained stable subcellular resolution imaging data (Figures 5B–D). Figure 5C shows the spontaneous Ca^2+^ transients of the dendrites, which were outlined with a black dashed rectangle in Figure 5B. Local Ca^2+^ transients are marked in red (Figure 5B), probably representing different information on the corresponding dendrites of the same neuron (Jia et al., 2010; Grienberger et al., 2015). Interestingly, some of the dendrites showed related Ca^2+^ transients evoked by sound stimuli (Figure 5D). Furthermore, Ca^2+^ transients at the single-spine level were also revealed (Figures 6A–C). An average image (from 1800 frames) shows a clear projection of a dendrite (Figure 6A). The spines of interest and corresponding dendritic shafts are indicated by arrowheads and rectangles, respectively. The spontaneous Ca^2+^ transients of these outlined ROIs in Figure 6A are shown in Figure 6B. Green color indicates the assembly of dendritic events, and red color indicates the single-spine inputs. Three consecutive trials of sound-evoked Ca^2+^ transients of these outlined ROIs are shown in Figure 6C.

### Chronic imaging over days in behaving mice

**Figure 7:**
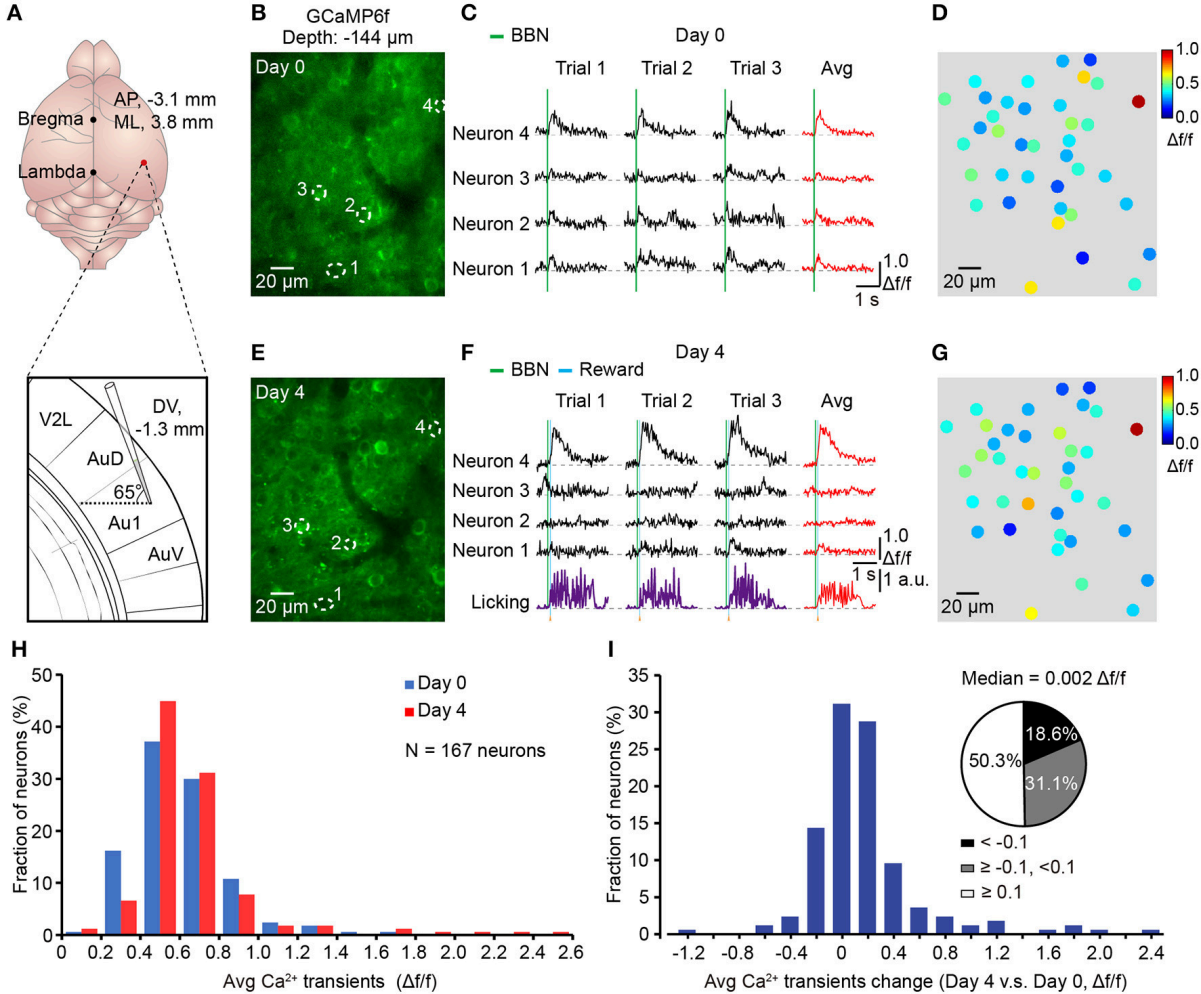
Chronic Ca^2+^ imaging in the Au1 throughout associative learning**. (A)** Upper, precise location of opening for virus injection. Lower, expanded view of titling electrode. **(B)** Average image (400 frames) of a focal plane in layer 2/3 of the Au1 before training (Day 0). **(C)** Sound-evoked Ca^2+^ transients from the neurons outlined with dashed circles in **B**. Three consecutive trials and their average in each neuron are shown. **(D)** Color mapping of the average amplitude of three consecutive trials evoked by the BBN for each neuron in **B** before training (Day 0). **(E)** Average image (400 frames) of the same focal plane after training (Day 4). **(F)** Sound-evoked Ca^2+^ transients from the same neurons after training. The simultaneous licking behaviors are also presented. **(G)** Color mapping of the average amplitude of three consecutive trials evoked by the BBN for each neuron after training (Day 4). **(H)** Distribution of the averaged response amplitudes of 3 consecutive trials in naive (Day 0) and conditioned (Day 4) states, respectively. *N* = 167 neurons from 4 mice. **(I)** Histogram shows the distribution of the change (Day 4 vs. Day 0) in the averaged Ca^2+^ amplitude of each neuron. Pie chat shows 50.3% neurons changed ≥0.1Δf/f (White), 18.6% neurons changed <-0.1Δf/f (Black) and the others between (Gray).

To reduce the chance of infection, we used a three-step method. First, the auditory cortex was labeled with GCaMP6f delivered from AuD by a tilted electrode to avoid direct damage to the Au1 (Figure 7A). Second, the head-post and chronic cranial window were implanted 2 weeks later. Third, two-photon imaging during associative learning tasks was applied 7–10 days after cranial window implantation to avoid inflammation and edema. Using this method, we successfully achieved chronic two-photon imaging in the Au1 of behaving mice (Figure 7). Activities of the viral labeled neurons were monitored for days during licking throughout behavioral training. Comparing the signals acquired under the naive state (Day 0, Figures 7B–D) to the trained state (Day 4, Figures 7E–G), neurons showed changed responses to the BBN stimuli (Figures 7B,E), which were the cues of the water reward and induced licking behavior afterwards (Figure 7F). Further comparison showed that the overall average amplitude was slightly increased (Figure 7H). For each neuron, change in the average response amplitude was presented (Figure 7I), and more than half of the neurons had a larger average response amplitude in the Day 4 than in the Day 0. The median value of all the changes in average amplitude was 0.002 Δf/f (Figure 7I).

## Discussion

### Summary of our method

We developed a protocol for *in vivo* two-photon Ca^2+^ imaging of the Au1 in behaving mice. This protocol included a custom-made head fixation apparatus that was able to achieve head fixation of the head-rotated mouse and minimize the brain motions. Water restriction was used to assist the habituation to head fixation and the associative learning of the sound-triggered licking task. After 6 training sessions, mice showed conditioned performance with head fixation. The conditioned mice were often still or sometimes grooming in the body tube. Minimizing motions between the brain and the objective would facilitate functional imaging using Ca^2+^ indicators in behaving mice (Dombeck et al., 2007; Greenberg and Kerr, 2009). The brain motions were apparent during online imaging, and the motions parallel with the focal plane (XY-plane) were mostly corrected offline (Supplementary Video 1). After correction, a relatively high signal-to-noise ratio was presented (Supplementary Figure 2), comparable to those from anesthetized mice (Li et al., 2017b). With the application of synthetic Ca^2+^ indicators, we were able to track the Ca^2+^ transients in the neuronal population (Figure 3), dendrites (Figure 5), and dendritic spines (Figure 6) in the auditory cortex of behaving mice. Moreover, the head fixation was stable enough for conducting loose patch, allowing simultaneous electrophysiology recordings (Figure 4) with two-photon Ca^2+^ imaging.

### Imaging and electrophysiology during sound-licking associative behavior

The major approach of neuroscience research is to link neural activities to behaviors. Thus, development of a simple and stable behavior associated with the perceptual cues in mice would be invaluable, as it would facilitate monitoring sensory responses during behavior. Our method of imaging neuronal populations in the Au1 demonstrated the feasibility of optically monitoring behavior-related neural activity at cellular resolution. For example, according to population imaging, a portion of neurons were activated by the BBNs (Figure 3D) and simultaneously with the licking behaviors (Figure 3B). Cell-attached recordings showed that most of Ca^2+^ transients were action potential related, and the single action-potential-evoked Ca^2+^ transients had an amplitude of approximately 0.4 Δf/f (Figure 4G), which was consistent with previous studies under anesthesia (Tada et al., 2014). Our method provides a possibility to monitor the neural responses over days. Using this method, one can compare the activities of single neurons or the same group of neurons at different time points. We showed an example in Figure 7, in which we performed chronic recordings over 5 days.

### Comparison to other methods

Two-photon imaging in the auditory cortex of behaving mice has been previously achieved by tilting the objective to fit the upright head-fixed mice (Kato et al., 2015; Kuchibhotla et al., 2017). Tilting the objective can reduce the difficulties in animal head fixation, and the two-photon imaging systems whose objective can be tilted are available commercially. With this procedure, one can easily perform motor behaviors like locomotion with the treadmill apparatus. In this study, our protocol provides an alternative way for two-photon imaging in the auditory cortex of behaving mice. First, our protocol is applicable for the conventional imaging systems whose objective could not be easily tilted. Second, with our procedure, the manipulation of the objective or of the electrode is simpler, which facilitates electrophysiological experiments, such as cell-attached recordings. Third, our recording chamber could be easily filled with liquid medium, such as ACSF. Fourth, frequently tilting the objective may result in drifting of the beam position, and this is not the case in our protocol. Although our protocol needs extra efforts in head fixation, this difficulty can be overcome by additional practices and the use of the custom-made head fixation apparatus (Figure 1). Like the treadmill and similar approaches (Dombeck et al., 2009, 2010; Marbach and Zador, in review) that increase animal comfort and allow stable two-photon imaging during locomotion, the body tube (Figure 1E) can also increase animal comfort through whole-body support and reduce unnecessary body movements through proper restraint. This design is particularly suitable for the licking behavior and provides a relatively stable cortical surface for imaging.

Previous studies have also used a head fixation strategy by rotating the mouse head to achieve two-photon imaging in the auditory cortex of awake mice (Issa et al., 2014; Deneux et al., 2016). By comparison, our method was optimized at several aspects. First, the head-post and the recording chamber were implanted separately. Thus, craniotomy could be performed at any time after head-post implantation, which made the skull above the Au1 intact during training sessions. Second, the head-post was cemented to the whole dorsal surface of the skull (Supplementary Figure 1) for tighter fixation, other than cemented around the cranial window. Third, because the head of the mouse was rotated ~70°, the upright objective could be directly aimed at the Au1 which was at horizontal level. However, in the previous study, the head of the mouse was rotated ~45° (Issa et al., 2014), which made the upright objective directly aimed at the junction of the AuD and the Au1. In this situation, extra efforts were needed to cover the whole Au1 region, such as a cranial window of ~5 mm in diameter (Deneux et al., 2016). Moreover, the custom-made head-post could be easily modified by adjusting the angle (Figure 1D) to reach other lateral brain regions. Fourth, the recording chamber (Figure 1C) with ACSF provided standard medium for electrophysiological experiments in behaving mice.

To investigate the precise neural activities during associative learning, a chronic imaging preparation was designed to repeatedly image the same group of neurons over days. By using genetically encoded Ca^2+^ indicators, such as AAV-GCaMP6f (Chen et al., 2013), our method could be applied to investigate the neuronal population dynamics in the Au1 throughout the process of associative learning, including the naive, conditioned and extinction states. In addition, our method provides an opportunity to investigate the micro-circuitry at the dendrite and spine levels in the Au1 of behaving mice. Both the electroporation (Nevian and Helmchen, 2007; Kitamura et al., 2008) and the viral injection (Chen et al., 2013) could be used to label the dendrites and dendritic spines. Furthermore, other dye-loading approaches, such as through intracellular electrodes (Svoboda et al., 1997; Helmchen et al., 1999), might provide similar dendritic labeling compared with our methods. Using AAV-GCaMP6, neurons and their dendrites could be sparsely labeled, which enables both cellular and sub-cellular imaging simultaneously. However, the labeled dendrites could form a bright background for imaging, and contaminate the Ca^2+^ transients within the region of the soma. For subcellular imaging, the electroporation technique provides a cleaner imaging background and the dendrites of the same neuron are labeled evenly (Kitamura et al., 2008).

### Future prospect

In general, the auditory cortex is less frequently studied with two-photon imaging because of its lateral location and the constraints of the use of the other research tools, such as electrophysiological recordings. Our protocol provides a simple method of the head-rotated head fixation, other than titling the objective, to facilitate two-photon imaging in the auditory cortex of behaving mice. It facilitates the application of the other tool box together with the two-photon imaging system for investigating the neuronal basis of audition-related behaviors.

Based on the combination of new imaging advances and genetic techniques, our protocol may achieve a wider application. For example, using a trapezoidal scanning method, simultaneously imaging neuronal populations within a 3D volume may be feasible (Andermann et al., 2010). By using GRIN lenses (Jung et al., 2004; Moretti et al., 2016) or far-infrared Ca^2+^ indicators, such as Cal-590 AM (Tischbirek et al., 2015), two-photon imaging in the deep brain of behaving mice is viable. Using the Cre-LoxP recombination system (Tsien et al., 1996), the activities of cell-type specific neurons can be monitored. Using optogenetic methods, the activities of cell type–specific neurons can be manipulated via the activation and inhibition of genetically encoded light-sensitive channels (Boyden et al., 2005; Deisseroth and Hegemann, 2017).

## Author contributions

RL, MW, JYan, XLi, and XC contributed to the design of the study and interpretation of the data; RL, MW, JYao, JZ, and XLi performed the experiments and acquired the data; RL, SL, MY, XLi, and XLiao processed and analyzed the data; RL, XLi, HJ, and XC wrote the manuscript with help from all the other authors.

### Conflict of interest statement

The authors declare that the research was conducted in the absence of any commercial or financial relationships that could be construed as a potential conflict of interest.

## Supplementary material

Supplementary Figure 1 The procedure of the head-post implantation. **(A)** Image of an anesthetized mouse on the surgery table in the stereotaxis instrument. **(B)** Hair removal using hair removal cream. **(C)** Wash with Betadine to prepare for skin removal. **(D)** Skin removal and tissue cleaning. **(E)** Adherence of the head-post.

Supplementary Figure 2 Comparison of the Ca^2+^ transients before and after image registration. **(A)** An averaged image (400 frames) from a behaving mouse. **(B)** The Ca^2+^ transients of 3 consecutive trials from 3 neurons (outlined in **A**) before registration. **(C)** The Ca^2+^ transients of 3 consecutive trials of 3 neurons (outlined in **A**) after registration.

Supplementary Figure 3 Comparison of the brain motion before and after habituation of head fixation. **(A–C)** The brain motions of mouse 1–3 before and after habituation. Red circles indicate the in plane (X–Y) movements (10 pixels = 4.5 μm). **(D)** Histogram of the brain motions that larger than 4.5 μm before and after habituation (*N* = 4 mice, 8 focal planes). The habituation reduced the brain motions with large amplitudes, especially those over 18 μm. Over 90% motions was less than 4.5 μm but not showed here. **(E)** Statistics shows significant reduction (^**^*p* = 0.008) of the brain motions that larger than 4.5 μm after habituation (*N* = 4 mice, 8 focuses). Extra experiments were done for this figure. We used 4 thy1-GFP mice. First, implantation of a head-post and a chronic cranial window over the Au1. Second, after 3-day's recovery, two-photon imaging of the naive state. Third, after 3-day's habituation, two-photon imaging of the habituated state.

Supplementary Video 1 Comparison of imaging data before and after motion correction. **(Left)** Video of a mouse performing the sound-triggered licking task. **(Middle)** Population imaging of L2/3 neurons in Au1 synchronized with the licking behavior of the mouse in the **Left**. The apparent X-Y axis motion artifacts were presented. **(Right)** Population imaging data of the **Middle** after motion correction.

Supplementary Table 1 The weights (g) of each mouse across training.

Supplementary File 1 3D print file for recording chamber.

Supplementary File 2 3D print file for head-post.

Supplementary File 3 3D print file for recording holder.

Supplementary File 4 3D print file for body tube.

Supplementary File 5 3D print file for head-post holder.
