# Supplementary figures and images for: Two-Photon Functional Imaging of the Auditory Cortex in Behaving Mice: From Neural Networks to Single Spines
Source: Front Neural Circuits. 2018 Apr 24;12:33. doi: 10.3389/fncir.2018.00033 (PMC5928246; doi:10.3389/fncir.2018.00033)

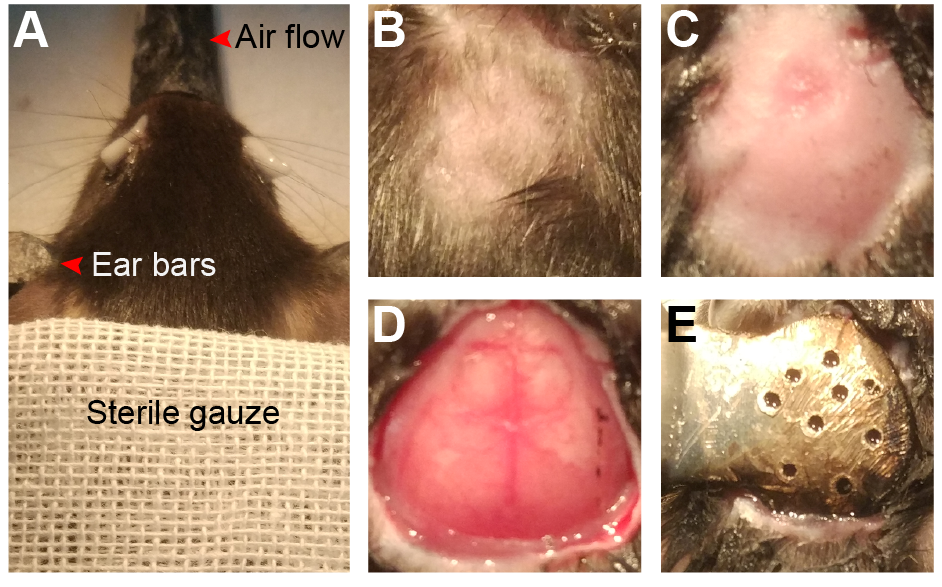

Supplement: Supplementary Figure 1 — The procedure of the head-post implantation. (A) Image of an anesthetized mouse on the surgery table in the stereotaxis instrument. (B) Hair removal using hair removal cream. (C) Wash with Betadine to prepare for skin removal. (D) Skin removal and tissue cleaning. (E) Adherence of the head-post. [file Image_1.TIF]

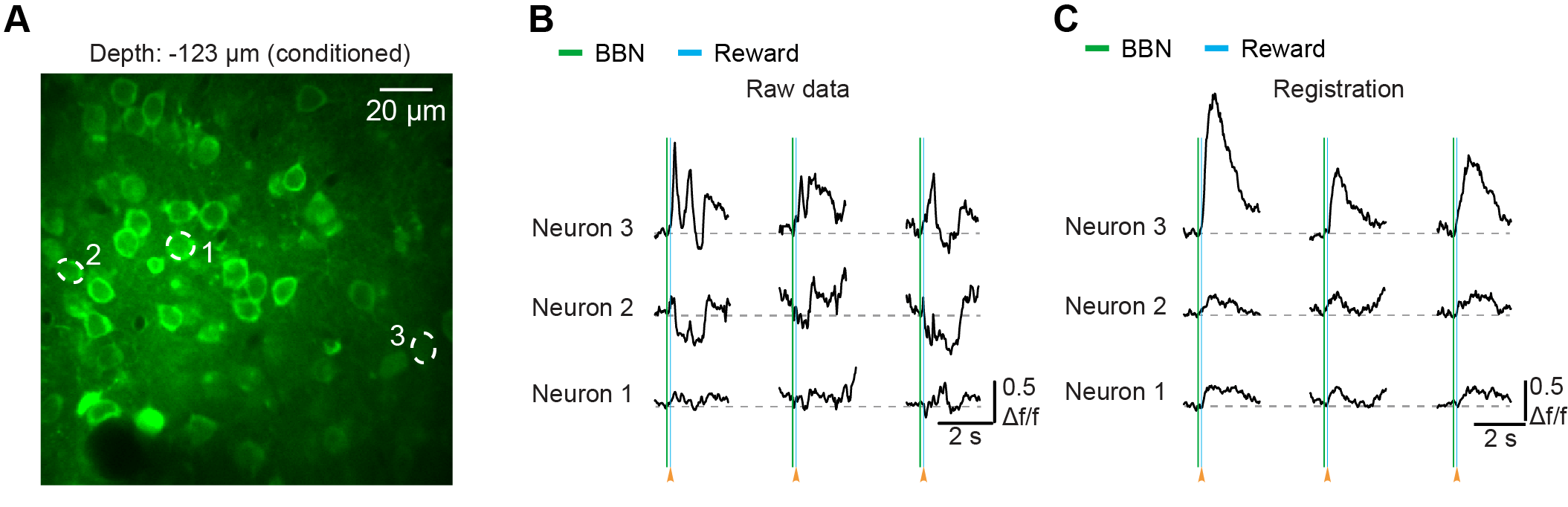

Supplement: Supplementary Figure 2 — Comparison of the Ca2+ transients before and after image registration. (A) An averaged image (400 frames) from a behaving mouse. (B) The Ca2+ transients of 3 consecutive trials from 3 neurons (outlined in A) before registration. (C) The Ca2+ transients of 3 consecutive trials of 3 neurons (outlined in A) after registration. [file Image_2.TIF]

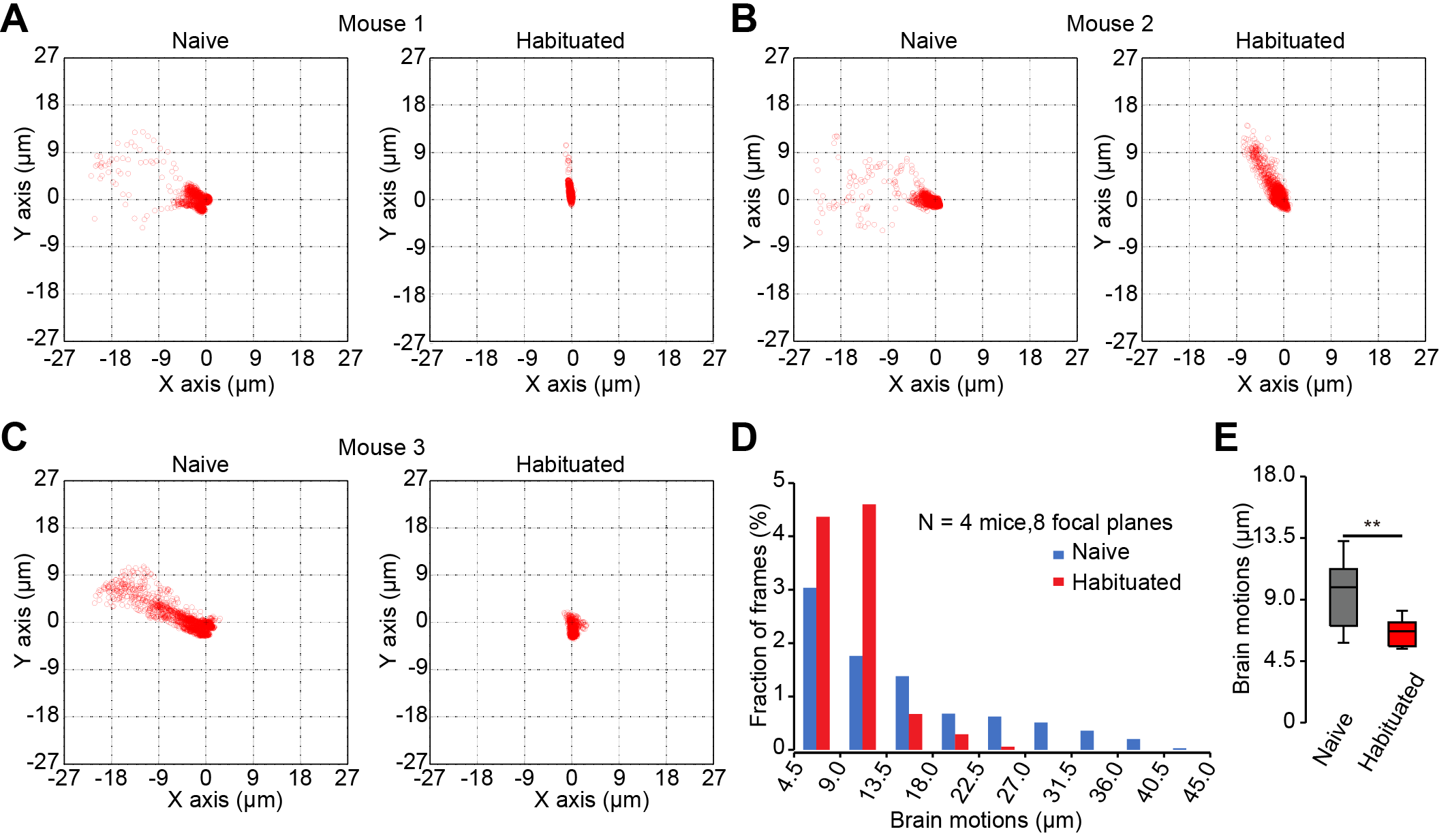

Supplement: Supplementary Figure 3 — Comparison of the brain motion before and after habituation of head fixation. (A–C) The brain motions of mouse 1–3 before and after habituation. Red circles indicate the in plane (X–Y) movements (10 pixels = 4.5 μm). (D) Histogram of the brain motions that larger than 4.5 μm before and after habituation (N = 4 mice, 8 focal planes). The habituation reduced the brain motions with large amplitudes, especially those over 18 μm. Over 90% motions was less than 4.5 μm but not showed here. (E) Statistics shows significant reduction (**p = 0.008) of the brain motions that larger than 4.5 μm after habituation (N = 4 mice, 8 focuses). Extra experiments were done for this figure. We used 4 thy1-GFP mice. First, implantation of a head-post and a chronic cranial window over the Au1. Second, after 3-day's recovery, two-photon imaging of the naive state. Third, after 3-day's habituation, two-photon imaging of the habituated state. [file Image_3.TIF]
