# Supplementary material for: Two-Photon Functional Imaging of the Auditory Cortex in Behaving Mice: From Neural Networks to Single Spines
Source: Front Neural Circuits. 2018 Apr 24;12:33. doi: 10.3389/fncir.2018.00033 (PMC5928246; doi:10.3389/fncir.2018.00033)
Supplement: Supplementary Table 1 — The weights (g) of each mouse across training. [file Table_1.DOCX]

**SUPPLEMENTARY TABLE 1 | The weights (g) of each mouse across training.**

| Tag. No. | Naive | Session1 | Session2 | Session3 | Session4 | Session5 | Session6 |
| --- | --- | --- | --- | --- | --- | --- | --- |
| 4925 | 22.4 | 20.1 | 18.6 | 18.9 | 19.1 | 19.6 | 18.7 |
| 4926 | 24.8 | 22.0 | 21.2 | 20.5 | 20.3 | 21.7 | 22.3 |
| 4927 | 31.2 | 28.3 | 26.8 | 27.4 | 26.8 | 28.5 | 27.2 |
| 4928 | 27.1 | 23.8 | 23.0 | 23.3 | 23.2 | 24.0 | 24.5 |
| 4929 | 23.9 | 20.8 | 19.9 | 19.8 | 19.4 | 21.2 | 21.6 |
| 4930 | 25.3 | 22.3 | 21.5 | 21.8 | 21.4 | 22.2 | 22.5 |
